# Society for the Study of Inebriety

**Published:** 1892-07-16

**Authors:** 


### HOSPITAL MEETINGS AND SOCIETIES.

### SOCIETY FOR THE STUDY OF INEBRIETY.

A meeting of the above Society was held at four p.m. on Tuesday, July 5th, in the rooms of the Medical Society of London, 11, Chandos Street, Cavendish Square. The chair was taken by the President of the Society, Dr. Norman Kerr.

Dr. Kerr explained that Dr. Williams had consented to postpone the reading of his paper until the October meeting, in order that they might discuss the Keeley "cure" for inebriety. He then referred to some of the methods employed in the treatment of intemperance, and amongst others he mentioned hypnotism, which he described as a straightforward and open remedy, and not subject to the reproach of being called a secret remedy. He then traced the history of the so-called "gold cures." The present "remedy" claimed to cure 100 per cent. of the cases in three weeks. Dr. Kerr then detailed the circumstances which had brought the present "cure" into prominence. He went on to say that if this "cure" was a genuine American temperance remedy it ought to have received the support of those men who were identified with the temperance movement in America, but such was not the case. The compound had been analysed for the Society by an eminent analyst, whose name Dr. Kerr was not at liberty to make public, but whose reputation was second to none in the United Kingdom. His analysis gave the following results: A bitter, probably Chiretta, mixed with a similar American compound; water, 61·31 per cent.; sugar, 6 per cent.; a small quantity of mineral salts, probably lime. There was also 27·55 per cent. of alcohol, and no gold or chlorides. This represented the analysis of bottle No. 1, the "remedy" consisting of bottles Nos. 1 and 2. Dr. Kerr concluded by saying that the compound had been condemned by the whole reputable press of America and Great Britain. The "remedy" was opposed to the whole principle of temperance reformation, for the reason that the basis of the treatment of intemperance consisted in cutting off the poison.

Dr. J. E. Usher gave an account of a visit he had made to Dr. Keeley's establishment at Dwight. He further said that the treatment was an expensive one.

The Chairman explained that Dr. Keeley had been unable to accept his invitation to attend the meeting owing to the fact that he had arranged to address a meeting that evening at St. James's Hall.

Dr. Morton proposed, "That this meeting of the Society for the Study of Inebriety, of which members are registered British medical practitioners, is of opinion that any so-called 'cures' for inebriety, the composition of which is not disclosed, are unfit to be commended by honourable members of the medical profession, who are bound to place the full details of their treatment before their professional colleagues—a requirement as essential in the interest of the public as it is consonant with the disinterested practice of scientific therapeutics."

This proposal was seconded by Dr. J. Smith, and was carried unanimously.

Surgeon-Major Pringle moved, "That this meeting having been informed by a competent London analyst, who has made a special analysis, that the alleged 'bichloride of gold cure' shows no trace of gold or chlorides, and contains 27·5 per cent. of alcohol, condemns unreservedly the prescription of such an intoxicating preparation to an inebriate."

This proposal was seconded by Mr. Jabez Hogg, and carried unanimously.

A vote of thanks to Dr. Usher and Dr. Norman Kerr, proposed by Dr. F. R. Lees and seconded by Dr. John Hilton, was carried unanimously, and the proceedings terminated.

